# A simple microplastic splitter for subsampling expanded polystyrene particles

**DOI:** 10.1016/j.mex.2023.102489

**Published:** 2023-11-17

**Authors:** Ryota Nakajima, Noriyuki Isobe, Nisha Singh

**Affiliations:** Japan Agency for Marine-Earth Science and Technology (JAMSTEC), 2-15 Natsushima Yokosuka, Kanagawa, Japan

**Keywords:** Microplastics, Splitting, Dividing, EPS, Plastic particles, Radial microplastic splitter

## Abstract

With the high number of microplastic-like particles captured by net hauls including manta or neuston nets, it is often required to subsample in order to decrease sample volume for microplastic enumeration and analysis. Plankton splitter is commonly used to divide microplastic samples. However, current devices such as Folsom plankton splitter and Motoda box splitter have accuracy issues in separating highly buoyant microplastics, namely expanded polystyrene (EPS) as they tend to adhere to the inner walls. Inspired by an apple cutter, we have developed a simple radial splitter made of stainless steel that efficiently divides EPS microplastic samples into precise aliquots. With this simple device, we uniformly divided EPS microplastic samples from marine environments into eight aliquots with no significant differences. The device is a versatile tool to partition all buoyant microplastics including polypropylene and polyethylene microplastics.

• The method developed facilitates the precise division of buoyant microplastics into equal aliquots.
• The method is specifically effective in splitting expanded polystyrene particles with high buoyancy.

## Graphical abstract

**Figure:**
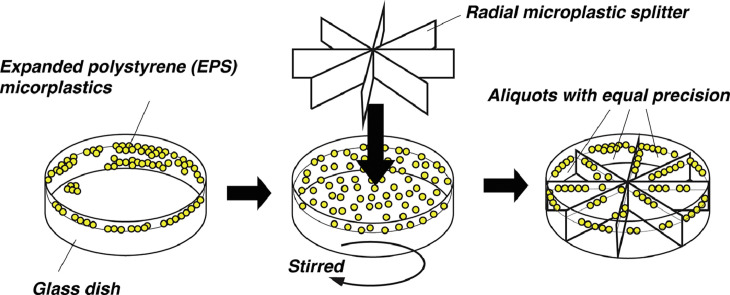

Specifications Table

**Table:** 

Subject area:	Environmental Science
More specific subject area:	Plastic pollution
Name of your method:	Radial microplastic splitter
Name and reference of original method:	No original method
Resource availability:	HDPE pellet (STANDARD), STP009–2030_01 https://standard-testpiece.com PP pellet (STANDARD), STP009–2030_03 https://standard-testpiece.com

**Method details**

## Background

Subsampling of microplastic (plastic particles < 5 mm) samples is often necessary to manage sample volume. This need arises due to the substantial quantities of microplastic-like particles that net hauls and sieves can capture [1], [2], [3], [4]. A notable instance can be observed in sea-surface samples collected by manta or neuston nets in coastal and estuarine waters often with a significant abundance of microplastics (>1000 particles per sample) [5,6]. This can interfere with the sorting and counting of microplastics given time and human constraints, compelling the need for subsampling. To address this, plankton splitters such as Folsom plankton splitter [7] and Motoda box splitter [8] are commonly used to divide microplastic samples. However, during the separation process, pouring divided media containing microplastics gets challenging with buoyant particles as they adhere onto the inner wall of splitters. This phenomenon is particularly pronounced for expanded polystyrene (EPS, often called Styrofoam), which is one of the commonly found types of microplastics in coastal waters [5,[9], [10], [11], [12], [13], [14], [15]. Due to the capillary force and surface free energy difference [16], EPS particles with higher buoyancy (or lower specific gravity, 0.01 – 0.04 g cm^−3^) rapidly adhere to the inner wall of the splitters. Consequently, it is impossible for the current plankton splitters to divide samples into equal aliquots with precision. In evaluating existing splitting devices, we identified the need for a device that allows simple and efficient subsampling of EPS microplastics. In this context, we report a simple subsampling device for EPS microplastic samples.

### Radial microplastic splitter

**Fig. 1:**
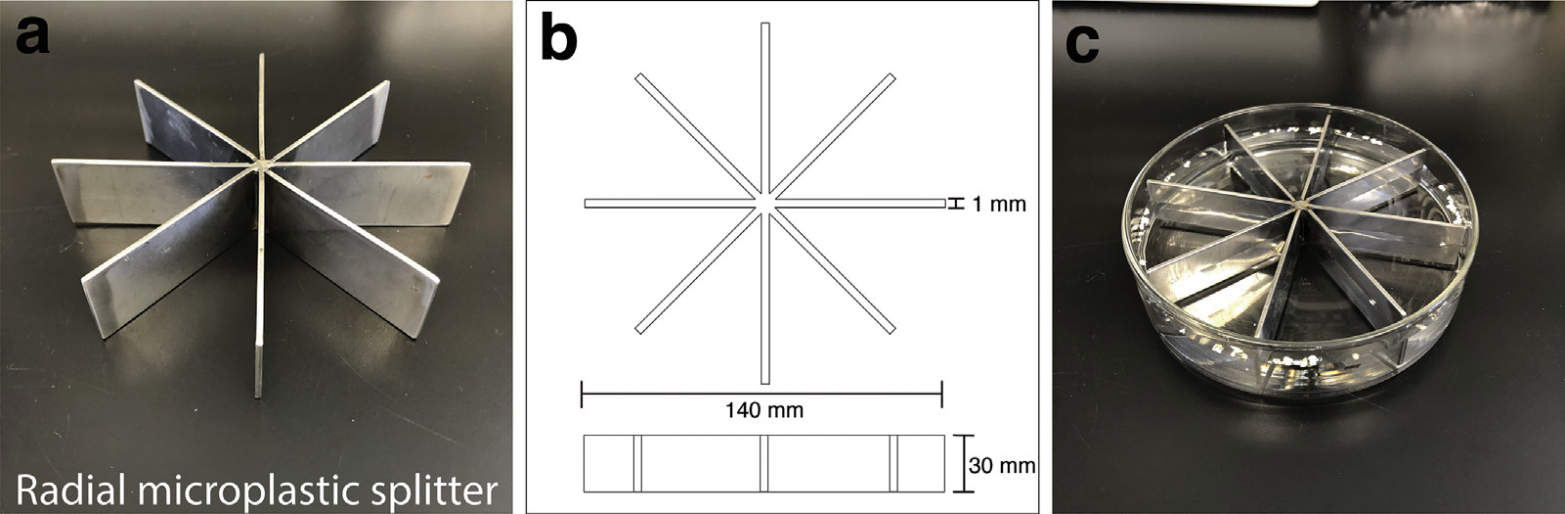
Radial microplastic splitter optimized for expanded polystyrene (EPS) microplastics samples: (a) the stainless-steel splitter has eight compartments, (b) schematic diagram of the splitter, (c) the splitter placed in a glass dish containing EPS particles from the marine environment.

We developed a simple splitter made of stainless-steel that can fit in a glass dish (petri dish), drawing inspiration from an apple cutter that can cut an apple into eight pieces in a single step (Fig. 1a). The radial microplastic splitter can divide floating EPS microplastics into eight aliquots in a single step. This splitter has a radial symmetry of 140 mm diameter and 30 mm height with eight equal compartments (Fig. 1b). A media containing microplastics is placed in a glass dish and is thoroughly stirred prior to separation. Once the water stops flowing and the microplastic particles have adhered to the wall, the radial splitter is placed into the glass dish so that the floating particles are divided into eight aliquots (Fig. 1c). Finally, the floating particles from desired number of aliquot are sucked with a glass pipette for subsampling. As long as the diameter of the radial splitter is a tight fit to the internal diameter of a glass dish, it prevents the intermixing of floating particles in each compartment when an aliquot is extracted.

### Method validation

To evaluate the separation accuracy of the radial splitter, first, we tested the splitter using virgin EPS particles with known concentrations. Secondly, we assessed the performance of the radial splitter using EPS particles collected from the marine environment. Finally, we investigated the radial splitter's effectiveness using particles of different polymer types including polyethylene (PE, 0.90–0.91 g cm^−3^) and polypropylene (PP, 0.90–0.91 g cm^−3^). We selected these three polymers as they represent the major polymer classes of buoyant microplastics in surface waters from various locations [17].

**Fig. 2:**
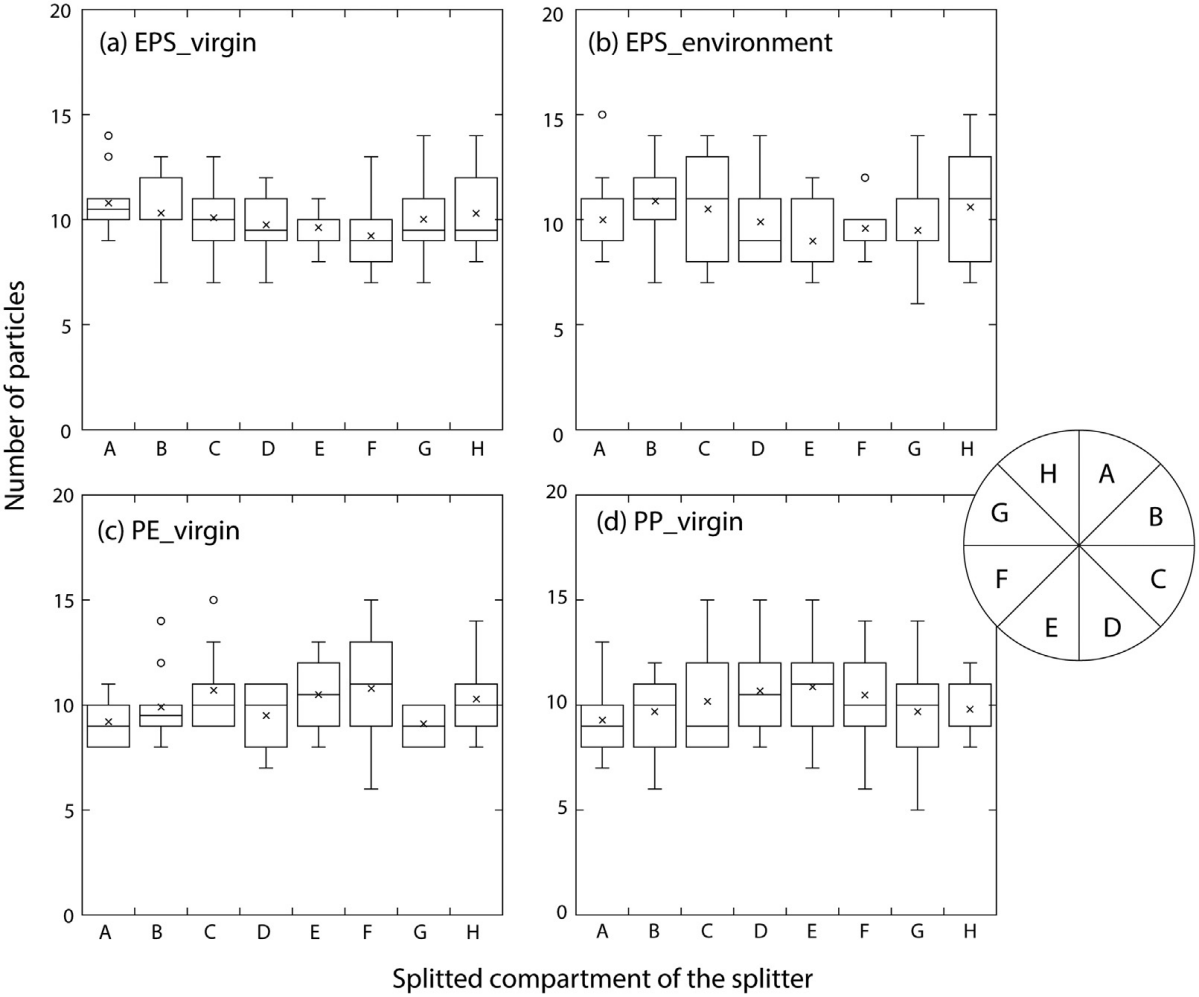
Boxplots of microplastic number collected in eight different compartments (A-H) with the radial microplastic splitter in (a) virgin expanded polystyrene (EPS) particles, (b) EPS particles from marine sample, (c) virgin polyethylene (PE) particles and (d) virgin polypropylene (PP) particles. The line in the middle of the boxes represents the median values; the tops and bottoms of the boxes denote the 75 % and 25 % quartiles, respectively, and the top and bottom of the error bars show the maximum and minimum values, excluding outliers (circles). Crosses denote the mean data.

For the first experiment, a batch of 80 virgin EPS particles (mean diameter 1.5 ± 0.15 mm, *n* = 20) were placed in the glass dish with seawater and stirred thoroughly. After placing the radial splitter, the number of particles in eight compartments of the splitter (here, describing compartments A to H, Fig. 2) was counted. This procedure was repeated ten times and the average (mean ± standard deviation, SD) number of particles in each compartment was recorded. Then the differences in the number of divided particles in each compartment (or aliquot) were determined using one-way ANOVA. The normality of the data and homogeneity of variance were examined and verified before ANOVA analysis using a chi-square test and a Bartlett test, respectively. A difference at *p*<0.05 was considered significant. The subsampling error in each aliquot was calculated as follows:

$$\text{Subsampling}\;\text{error}=\frac{\text{number}\;\text{of}\;\text{particles}\;\text{in}\;a\;\text{compartment}\;\times \;\text{number}\;\text{of}\;\text{compartment}\;\left(8\right)}{\text{total}\;\text{number}\;\text{of}\;\text{particles}\;\left(80\right)}$$

The coefficient of variation (CV in%) of the subsampling error for each aliquot was calculated for the purpose of comparison with other studies:

$$\text{Coefficient}\;\text{of}\;\text{variation}\;\left(\text{CV}\right)=\frac{\text{standard}\;\text{deviation}}{\text{mean}}\times 100$$

For the next phase, we prepared a batch of 80 EPS particles collected by a neuston net in Sagami Bay, Japan in September 2019. Further, details regarding the sampling of the natural EPS particles are described elsewhere [5]. These environmental EPS particles (mean diameter 2.6 ± 0.58 mm, *n* = 20) were placed in the glass dish with seawater and subjected to the same process as described above. Finally, 80 virgin PE (3.4 ± 0.20 mm, *n* = 20) and virgin PP (4.1 ± 0.09 mm, *n* = 20) pellets were investigated using the radial splitter unit as described above.

Fig. 2 shows the results of the particle numbers in each compartment from the first, second, and third experiments. The raw values are available in the supplementary information (Table S1). Mean (± SD) numbers of particles in different compartments A-H ranged from 9.2 (± 1.8) to 10.8 (± 1.6) particles for virgin EPS (overall mean: 10.0 ± 0.50 particles), 9.0 (± 1.8) to 10.9 (± 2.2) particles for environmental EPS (overall mean: 10.0 ± 0.64 particles), 9.1 (± 0.9) to 10.8 (± 2.9) particles for virgin PE (overall mean: 10.0 ± 0.67 particles), and 9.2 (± 1.8) to 10.8 (± 2.6) particles for virgin PP (overall mean: 10.0 ± 0.56 particles).

No significant statistical differences were found in the numbers among eight aliquots of the radial splitter for all tested samples i.e., virgin EPS (*p* = 0.589), environmental EPS (*p* = 0.546), virgin PE (*p* = 0.191), and virgin PP (*p* = 0.731). The result indicates efficient microplastic dividing using the radial splitter for both virgin and natural samples and across the three polymer types.

The subsampling errors (mean ± SD) in compartments A-H ranged from 0.9 ± 0.1 to 1.1 ± 0.3 for all polymer types (Table 1). The CVs varied from 15.0 % to 21.6 % (overall mean: 17.3 ± 3.3 %) for virgin EPS, 14.9 % to 26.0 % (overall mean: 21.4 ± 3.5 %) for environmental EPS, 9.6 % to 26.5 % (overall mean: 16.4 ± 5.2 %) for virgin PE, and 15.4 % to 27.9 % (overall: 22.0 ± 4.2 %) for virgin PP. When dividing the samples into two aliquots with the radial splitter, i.e., by pooling microplastic particles from four compartments (A-D) in one aliquot and four compartments (E-H) into another, the CVs for each aliquot would be as follows: 7.1 % to 7.5 % for virgin EPS, 8.8 % to 9.4 % for environmental EPS, 8.3 % to 8.6 % for virgin PE, and 8.3 % to 8.5 % for virgin PP. Furthermore, when dividing the samples into four aliquots with our device, i.e., by pooling microplastic particles from two compartments (A-B, C-D, E-F, and G-H), the CVs for each four aliquot would be 9.5–15.9 % for virgin EPS (overall: 12.6 ± 2.6 %), 10.8–15.1 % for environmental EPS (overall: 12.5 ± 1.8 %), 9.8–16.9 % for virgin PE (overall: 12.8 ± 3.0 %) and 12.3–17.8 % for virgin PP (overall: 15.5 ± 2.3 %).

**Table 1:** Subsampling errors and coefficient of variations (CVs in%) using the radial microplastic splitter. A to H denote the eight compartments of the splitter. The error values represent ten replicate measurements from different compartments A-H shown in Fig. 2. EPS, expanded polystyrene; PE, polyethylene; PP, polypropylene.

EPS_virgin											Mean	SD	CV%		
A	1	1.1	1	1.1	1.3	0.9	0.9	1	1.1	1.4	1.1	0.2	15.0		
B	1	1.2	1	0.9	1.3	1.2	1	0.7	1	1	1.0	0.2	16.5		
C	1.1	1.1	0.9	1	1	1	1.3	1.1	0.9	0.7	1.0	0.2	15.8		
D	0.9	0.7	0.9	0.7	1.1	1.2	0.9	1	1.2	1.1	1.0	0.2	18.9		
E	0.9	0.9	1	0.8	0.8	1	1.1	1	1	1.1	1.0	0.1	11.2		
F	0.9	0.7	1.3	0.9	0.9	0.7	1	0.9	0.8	1.1	0.9	0.2	19.7		
G	1.3	0.9	0.9	1.4	0.7	1	0.9	1	1.1	0.8	1.0	0.2	21.6		
H	0.9	1.4	1	1.2	0.9	1	0.9	1.3	0.9	0.8	1.0	0.2	19.4		
Overall mean ± SD													17.3	±	3.3
A-D	1	1.025	0.95	0.925	1.175	1.075	1.025	0.95	1.05	1.05	1.0	0.1	7.1		
E-H	1	0.975	1.05	1.075	0.825	0.925	0.975	1.05	0.95	0.95	1.0	0.1	7.5		
Median													7.3		
A-B	1	1.15	1	1	1.3	1.05	0.95	0.85	1.05	1.2	1.1	0.1	12.3		
C-D	1	0.9	0.9	0.85	1.05	1.1	1.1	1.05	1.05	0.9	1.0	0.1	9.5		
E-F	0.9	0.8	1.15	0.85	0.85	0.85	1.05	0.95	0.9	1.1	0.9	0.1	12.7		
G-H	1.1	1.15	0.95	1.3	0.8	1	0.9	1.15	1	0.8	1.0	0.2	15.9		
Overall mean ± SD													12.6	±	2.6

EPS_environment											Mean	SD	CV%		

A	1.5	0.9	0.8	1.2	0.9	0.9	0.9	1	1.1	0.8	1.0	0.2	21.6		
B	1	0.7	1	1.1	1.1	1.2	1.2	1.3	0.9	1.4	1.1	0.2	18.6		
C	1.2	0.8	0.8	1.3	0.8	1.3	1.4	1	0.7	1.2	1.1	0.3	24.7		
D	0.9	1.3	1.4	0.8	1	0.9	0.8	0.9	0.8	1.1	1.0	0.2	21.5		
E	1.1	1.2	0.8	0.7	0.8	0.7	1	0.8	1.1	0.8	0.9	0.2	20.3		
F	0.8	0.9	1.2	0.8	0.9	1	0.9	1.2	1	0.9	1.0	0.1	14.9		
G	0.6	0.9	0.9	0.9	1.4	0.7	1.1	1.1	0.9	1	1.0	0.2	23.4		
H	0.9	1.3	1.1	1.2	1.1	1.3	0.7	0.7	1.5	0.8	1.1	0.3	26.0		
Overall mean ± SD													21.4	±	3.5
A-D	1.15	0.925	1	1.1	0.95	1.075	1.075	1.05	0.875	1.125	1.0	0.1	8.8		
E-H	0.85	1.075	1	0.9	1.05	0.925	0.925	0.95	1.125	0.875	1.0	0.1	9.4		
Median													9.1		
A-B	1.25	0.8	0.9	1.15	1	1.05	1.05	1.15	1	1.1	1.0	0.1	12.4		
C-D	1.05	1.05	1.1	1.05	0.9	1.1	1.1	0.95	0.75	1.15	1.0	0.1	11.8		
E-F	0.95	1.05	1	0.75	0.85	0.85	0.95	1	1.05	0.85	0.9	0.1	10.8		
G-H	0.75	1.1	1	1.05	1.25	1	0.9	0.9	1.2	0.9	1.0	0.2	15.1		
Overall mean ± SD													12.5	±	1.8

PE_virgin											Mean	SD	CV%		

A	0.9	0.8	0.8	1	0.9	1	1.1	1	0.8	0.9	0.9	0.1	11.2		
B	0.9	0.8	1	0.9	0.8	1	0.9	1.2	1	1.4	1.0	0.2	18.7		
C	1	1.3	1	1.1	0.9	1.5	0.9	1.1	0.9	1	1.1	0.2	18.2		
D	0.7	1.1	0.8	1	1	1	0.9	0.8	1.1	1.1	1.0	0.1	15.1		
E	1.2	1.1	1.1	1	1.3	0.8	1.2	0.9	1	0.9	1.1	0.2	15.1		
F	1.3	0.9	1.5	0.9	0.8	0.6	1.2	1.2	1.4	1	1.1	0.3	26.5		
G	1	1	0.8	1	0.9	0.9	0.9	0.8	1	0.8	0.9	0.1	9.6		
H	1	1	1	1.1	1.4	1.2	0.9	1	0.8	0.9	1.0	0.2	16.5		
Overall mean ± SD													16.4	±	5.2
A-D	0.875	1	0.9	1	0.9	1.125	0.95	1.025	0.95	1.1	1.0	0.1	8.6		
E-H	1.125	1	1.1	1	1.1	0.875	1.05	0.975	1.05	0.9	1.0	0.1	8.3		
Median													8.4		
A-B	0.9	0.8	0.9	0.95	0.85	1	1	1.1	0.9	1.15	1.0	0.1	11.4		
C-D	0.85	1.2	0.9	1.05	0.95	1.25	0.9	0.95	1	1.05	1.0	0.1	13.0		
E-F	1.25	1	1.3	0.95	1.05	0.7	1.2	1.05	1.2	0.95	1.1	0.2	16.9		
G-H	1	1	0.9	1.05	1.15	1.05	0.9	0.9	0.9	0.85	1.0	0.1	9.8		
Overall mean ± SD													12.8	±	3.0

PP_virgin											Mean	SD	CV%		

A	0.9	0.8	1	1.3	0.7	1	0.9	0.7	1	0.9	0.9	0.2	19.0		
B	1.2	0.6	0.8	1.1	1	1.1	1.1	0.9	1	0.8	1.0	0.2	19.1		
C	0.8	0.9	1.5	0.9	1.4	1	0.8	1.2	0.8	0.8	1.0	0.3	26.2		
D	1.3	1.1	0.9	0.9	1.1	0.8	1.2	0.8	1.5	1	1.1	0.2	21.4		
E	1	1.5	0.8	1.1	0.7	0.9	1.1	1.3	1.2	1.2	1.1	0.2	22.2		
F	1.4	1.4	1	0.6	0.9	1.2	1.2	0.8	0.9	1	1.0	0.3	24.9		
G	0.5	0.9	0.8	1.1	1.4	1.1	0.8	1.1	0.7	1.2	1.0	0.3	27.9		
H	0.9	0.8	1.2	1	0.8	0.9	0.9	1.2	0.9	1.1	1.0	0.1	15.4		
Overall mean ± SD													22.0	±	4.2
A-D	1.05	0.85	1.05	1.05	1.05	0.975	1	0.9	1.075	0.875	1.0	0.1	8.5		
E-H	0.95	1.15	0.95	0.95	0.95	1.025	1	1.1	0.925	1.125	1.0	0.1	8.3		
Median													8.4		
A-B	1.05	0.7	0.9	1.2	0.85	1.05	1	0.8	1	0.85	0.9	0.1	15.6		
C-D	1.05	1	1.2	0.9	1.25	0.9	1	1	1.15	0.9	1.0	0.1	12.3		
E-F	1.2	1.45	0.9	0.85	0.8	1.05	1.15	1.05	1.05	1.1	1.1	0.2	17.8		
G-H	0.7	0.85	1	1.05	1.1	1	0.85	1.15	0.8	1.15	1.0	0.2	16.2		
Overall mean ± SD													15.5	±	2.3

In the previous study, the CV of the Folsom plankton splitter was examined using microplastic samples collected from a coastal environment primarily consisting of PE and PP particles (94 % of total particles) [2]. The finding of the study demonstrates that when samples were divided into two aliquots (ca. 8.5 % with a parameter of 297–1505) [2], it was comparable with the CVs obtained in the present study for two similarly divided aliquots (7.1 %−9.4 % with a parameter of 80). The previous study also acknowledged the tendency for the error to increase when the splitting was repeated. When splitting a microplastic sample into four aliquots with Folsom splitter the reported CVs were approximately 15 %−17 % [2]. The CVs obtained with our splitter, also using four aliquots (overall mean 12.5 %−15.5 %), were comparable with or slightly lower than those of Folsom splitter, indicating our radial splitter is tantamount to Folsom plankton splitter for dividing floating microplastics. Furthermore, the CVs of our splitter with a higher number of aliquot (i.e., eight aliquots, overall mean 16.4 %−22.0 %) were found to be comparable to or slightly higher than those of Folsom splitter with four aliquots (15 %−17 %). These comparisons conclude that our radial splitter is a suitable alternative to the Folsom plankton splitter.

Although the CVs in our device is similar to that of the previous method (i.e., Folsom plankton splitter) it is important to note that the primary focus was PE and PP microplastics in the previous study [2], and did not take into account EPS particles. The splitter in the current study offers the advantage of effectively and equally partitioning EPS particles. Currently, the Folsom plankton splitter and the Motoda box splitter are common devices for subsampling microplastics that can be collected by net hauls or other sampling devices [2,^18^,19]. However, when splitting EPS particles, rapid adhesion of EPS particles to the inside wall of the splitter due to high buoyancy (or low specific gravity) and surface free energy difference [16] is a significant challenge with the previous devices. As the particles remain adhered to the wall, pouring the media from the splitter ultimately results in uneven aliquots. More specifically, EPS particles stick to the wall surface of the plankton splitter before transferring all the particles into the splitting sections, which inhibits the “splitting” itself. EPS particles often dominate microplastic samples in coastal waters, especially after storms and/or near aquaculture farms that use EPS-made gears [5]. Given the substantial number of EPS microplastic particles in a single sample, subsampling is required to reduce sample volume for rapid estimation of the abundance. Applying water repellent onto the inner wall of plankton splitters may help to avoid buoyant particles being adhered to, but it may not be appropriate for further analysis of adsorbed pollutants and other chemicals such as additives on/within microplastics [20], [21], [22], [23]. In our device, while EPS particles may also stick to the glass wall surface, the even distribution to the inner walls of the round glass dish is achieved thorough stirring. This, in turn, enables equal splitting of the EPS particles when the radial splitter is subsequently inserted. Thus, the use of our radial splitter is more promising since it ensures the subsampling of EPS particles with precision irrespective of adhesion properties.

The radial splitter is equally efficient for splitting PE and PP microplastics, making it suitable for splitting all buoyant microplastics. Although we tested the radial splitter with a relatively small number of microplastics in this study, the splitter can also be used for a large number of microplastics. However, it should be noted that the radial splitter has space between the bottoms of the splitter and the glass dish, thus less buoyant microplastics such as polyvinyl chloride and polyethylene terephthalate particles can move together with water between the compartments and are not compatible with our splitter.

## Conclusion

We have developed a simple microplastic splitter, drawing inspiration from an apple cutter. This radial microplastic splitter is easy to use and facilitates rapid subsampling of floating microplastics, particularly EPS particles, in a single step. The radial splitter offers the advantage of subsampling EPS microplastics that would otherwise adhere to the inner wall of the previous splitters, resulting in uneven partitioning of aliquots.

## Ethics statements

No human, animal, and social media platforms were used in this study.

## Supplementary material *and/or* additional information

Data supporting the findings of this study are available in the supplementary material file provided along with this article.

## CRediT authorship contribution statement

**Ryota Nakajima:** Conceptualization, Formal analysis, Investigation, Data curation, Methodology, Validation, Visualization, Writing – original draft, Writing – review & editing. **Noriyuki Isobe:** Investigation, Methodology, Writing – review & editing. **Nisha Singh:** Methodology, Writing – review & editing.

## Declaration of Competing Interest

The authors declare that they have no known competing financial interests or personal relationships that could have appeared to influence the work reported in this paper.

## Data Availability

All data are available in this paper and Table S1.

## Appendix Supplementary materials

Image, application 1

## References

[1] McEachern K., Alegria H., Kalagher A.L., Hansen C., Morrison S., Hastings D. (2019). Microplastics in Tampa Bay, Florida: abundance and variability in estuarine waters and sediments. Mar. Pollut. Bull..

[2] Michida Y., Chavanich S., Chiba S., Cordova M.R., Cozsar Cabanas A., Glagani F., Hagmann P., Hinata H., Isobe A., Kershaw P., Kozlovskii N., Li D., Lusher A.L., Marti E., Mason S.A., Mu J., Saito H., Shim W.J., Syakti A.D., Takada H., Thompson R., Tokai T., Uchida K., Vasilenko K., Wang J. (2019). Guidelines for Harmonizing Ocean Surface Microplastic Monitoring Methods.

[3] Di Mauro R., Kupchik M.J., Benfield M.C. (2017). Abundant plankton-sized microplastic particles in shelf waters of the northern Gulf of Mexico. Environ. Pollut..

[4] Parmar S., Arbuckle-Keil G., Kumi G., Fahrenfeld N. (2023). Urban stormwater microplastic size distribution and impact of subsampling on polymer diversity. Environ. Sci. Processes Impacts.

[5] Nakajima R., Miyama T., Kitahashi T., Isobe N., Nagano Y., Ikuta T., Oguri K., Tsuchiya M., Yoshida T., Aoki K. (2022). Plastic after an extreme storm: the typhoon-induced response of micro-and mesoplastics in coastal waters. Front. Mar. Sci..

[6] Piehl S., Mitterwallner V., Atwood E.C., Bochow M., Laforsch C. (2019). Abundance and distribution of large microplastics (1–5mm) within beach sediments at the Po River Delta, Northeast Italy. Mar. Pollut. Bull..

[7] McEwen G., Johnson M., Folsom T.R. (1954). A statistical analysis of the performance of the Folsom plankton sample splitter, based upon test observations. Arch. fur Meteorol. Geophys. Bioklimatol. A.

[8] Motoda S. (1959). Devices of simple plankton apparatus. Mem. Fac. Fish. Hokkaido Univ..

[9] Eo S., Hong S.H., Song Y.K., Lee J., Lee J., Shim W.J. (2018). Abundance, composition, and distribution of microplastics larger than 20μm in sand beaches of South Korea. Environ. Pollut..

[10] Song Y.K., Hong S.H., Eo S., Han G.M., Shim W.J. (2020). Rapid production of micro-and nanoplastics by fragmentation of expanded polystyrene exposed to sunlight. Environ. Sci. Technol..

[11] Chan H.H.S., Not C. (2023). Variations in the spatial distribution of expanded polystyrene marine debris: are Asian’s coastlines more affected?. Environ. Adv..

[12] Kang J.H., Kwon O.Y., Lee K.W., Song Y.K., Shim W.J. (2015). Marine neustonic microplastics around the Southeastern Coast of Korea. Mar. Pollut. Bull..

[13] Nabizadeh R., Sajadi M., Rastkari N., Yaghmaeian K (2019). Microplastic pollution on the persian gulf shoreline: a case study of Bandar Abbas city, Hormozgan Province, Iran. Mar. Pollut. Bull..

[14] Cordova M.R., Purwiyanto A.I.S., Suteja Y. (2019). Abundance and characteristics of microplastics in the Northern Coastal waters of Surabaya, Indonesia. Mar. Pollut. Bull..

[15] Garcés-Ordóñez O., Espinosa L.F., Costa Muniz M., Salles Pereira L.B., Meigikos dos Anjos R. (2021). Abundance, distribution, and characteristics of microplastics in coastal surface waters of the Colombian Caribbean and Pacific. Environ. Sci. Pollut. Res..

[16] Zamani F., Ullah A., Akhondi E., Tanudjaja H.J., Cornelissen E.R., Honciuc A., Fane A.G., Chew J.W. (2016). Impact of the surface energy of particulate foulants on membrane fouling. J. Memb. Sci..

[17] Kim I.S., Chae D.H., Kim S.K., Choi S., Woo S.B. (2015). Factors influencing the spatial variation of microplastics on high-tidal coastal beaches in Korea. Arch. Environ. Contam. Toxicol..

[18] Lam T.W.L., Fok L., Lin L., Xie Q., Li H.X., Xu X.R., Yeung L.C. (2020). Spatial variation of floatable plastic debris and microplastics in the Pearl River Estuary, South China. Mar. Pollut. Bull..

[19] de Mendonça S.N., MacIsaac K., Moore A.M., Johnson C.L. (2021).

[20] Jang M., Shim W.J., Han G.M., Rani M., Song Y.K., Hong S.H. (2017). Widespread detection of a brominated flame retardant, hexabromocyclododecane, in expanded polystyrene marine debris and microplastics from South Korea and the Asia-Pacific coastal region. Environ. Pollut..

[21] Pan Y.F., Liu S., Lin L., Cheng Y.Y., Hou R., Li H.X., Yuan Z., Xu X.R. (2022). Release behaviors of hexabromocyclododecanes from expanded polystyrene microplastics in seawater and digestive fluids. Gondwana Res..

[22] Twyford S.I., Turner A. (2023). Association of metals with expanded polystyrene in the marine environment. Sci. Total Environ..

[23] Zhang H., Zhou Q., Xie Z., Zhou Y., Tu C., Fu C., Mi W., Ebinghaus R., Christie P., Luo Y. (2018). Occurrences of organophosphorus esters and phthalates in the microplastics from the coastal beaches in North China. Sci. Total Environ..

